# Impact of Kidney Function on Physiological Assessment of Coronary Circulation

**DOI:** 10.31083/j.rcm2510358

**Published:** 2024-10-08

**Authors:** Wojciech Zasada, Barbara Zdzierak, Tomasz Rakowski, Beata Bobrowska, Agata Krawczyk-Ożóg, Sławomir Surowiec, Stanisław Bartuś, Andrzej Surdacki, Artur Dziewierz

**Affiliations:** ^1^Clinical Department of Cardiology and Cardiovascular Interventions, University Hospital, 30-688 Krakow, Poland; ^2^KCRI, 30-347 Krakow, Poland; ^3^2nd Department of Cardiology, Institute of Cardiology, Jagiellonian University Medical College, 30-688 Krakow, Poland; ^4^Department of Anatomy, HEART-Heart Embryology and Anatomy Research Team, Jagiellonian University Medical College, 31-034 Krakow, Poland

**Keywords:** borderline stenoses, fractional flow reserve, glomerular filtration rate, instantaneous wave-free ratio, physiological assessment, resting full-cycle ratio

## Abstract

**Background:**

Diagnosing myocardial ischemia in chronic kidney disease (CKD) patients is crucial since coronary artery disease (CAD) forms the predominant cause of mortality in these patients. Thus, this study aimed to assess the impact of kidney function on the results of coronary circulation physiological assessment.

**Methods:**

Data were collected from 279 consecutive patients admitted to the Clinical Department of Cardiology and Cardiovascular Interventions at the University Hospital in Krakow. A total of 417 vessels were assessed for fractional flow reserve (FFR) and non-hyperemic resting pressure ratios, such as instantaneous wave-free ratio (iFR) and resting full-cycle ratio (RFR). Patients were categorized into two groups: glomerular filtration rate (GFR)-L (estimated GFR (eGFR) <70 mL/min/1.73 m^2^) and GFR-H (eGFR ≥70 mL/min/1.73 m^2^).

**Results:**

A total of 118 patients (42.3%) were included in the GFR-L group, while 161 patients (57.7%) were in the GFR-H group. The left anterior descending branch of the left coronary artery (LAD) was the assessed vessel in approximately 60% of procedures, the frequency of which was very similar in both study groups. Focusing solely on LAD assessments, both FFR metrics (continuous and binary) were comparable between the groups. In contrast, for non-LAD vessels, the GFR-H group revealed substantially reduced FFR values, with more vessels displaying significant constriction. Patients in the GFR-H group showed higher instances of FFR+ | iFR/RFR- discrepancies than their lower eGFR counterparts. An eGFR of 70 mL/min/1.73 m^2^ was the optimal cut-off to differentiate patients concerning the mentioned discrepancies.

**Conclusions:**

Kidney function influenced the coronary circulation physiological assessment results. Patients with reduced eGFR tended to have negative hyperemic assessments, especially in non-LAD vessels.

## 1. Introduction

Chronic kidney disease (CKD) continues to be a significant health concern. Even with recent advancements and more accessible treatments, it remains linked to a heightened risk of mortality and organ complications. This risk escalates as the disease progresses and kidney function deteriorates [1]. It is estimated that advanced CKD can curtail life expectancy by approximately 25 years [2]. Alarmingly, young patients under 35 with end-stage renal disease have an annual mortality rate that is about 500–1000 times higher than for their healthy counterparts of the same age and is similar to that for 85-year-olds in the general population [3].

An interesting observation arises from a closer examination of mortality causes among CKD patients. The likelihood of progression to end-stage renal disease requiring dialysis and subsequently dying during this stage is ten times rarer than dying prematurely before reaching a disease stage that necessitates dialysis [4]. Chronic kidney disease is a paramount risk factor for coronary artery disease (CAD). The danger of developing CVD surpasses the risk of CKD advancing to its end stage [5]. Concurrently, a reduction in the glomerular filtration rate (GFR) to values below 75 mL/min/1.73 m^2^ amplifies the cardiovascular mortality risk, making it notably higher than in the population with normal kidney function [6].

Since CAD is the predominant mortality cause in CKD patients [7], diagnosing myocardial ischemia is crucial. Previous studies suggest that CKD considerably heightens the likelihood of invasively diagnosing ischemic heart disease [8] and adversely impacts the long-term outcomes of percutaneous treatment [9]. Therefore, precision and vigilance are required when qualifying CKD patients for coronary angiography and accurately interpreting angiographic outcomes, potential simultaneous physiological evaluations of observed lesions, and invasive treatments. Thus, this study aimed to assess the impact of kidney function on the results of coronary circulation physiological assessment.

## 2. Materials and Methods

**Fig. 1.**
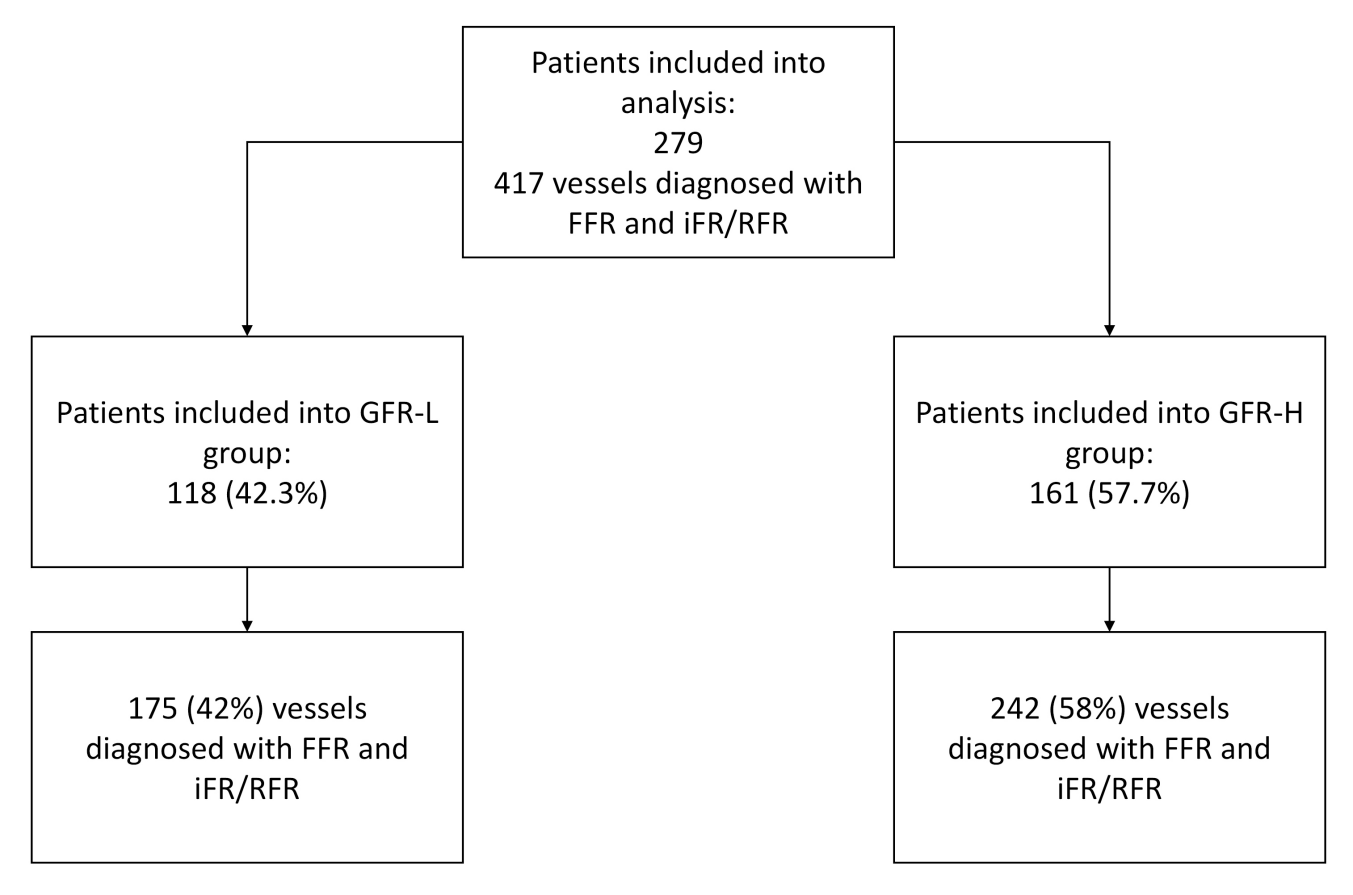
**Study flowchart**. FFR, fractional flow reserve; (e)GFR, (estimated) glomerular filtration rate; iFR, instantaneous wave-free ratio; RFR, resting full-cycle ratio; L, indicates the group with an eGFR <70 mL/min/1.73 m^2^; H, indicates the group with an eGFR ≥70 mL/min/1.73 m^2^.

A retrospective analysis was conducted on consecutive patients diagnosed with stable coronary artery disease who were admitted to the Clinical Department of Cardiology and Cardiovascular Interventions at the University Hospital in Krakow from 2020 to 2021. These patients underwent invasive physiological assessment of their coronary circulation due to the presence of borderline atherosclerotic lesions, defined based on visual evaluation of angiography by the operator as 50–90% diameter stenosis. All patients with stable coronary artery disease who underwent physiological assessment of borderline changes in at least one coronary artery using both the hyperemic method (FFR) and the non-hyperemic method (iFR or RFR) were included in the analysis. We did not define exclusion criteria. Patients were stratified into two subgroups based on their estimated GFR (eGFR), which was assessed upon admission using the Modification of Diet in Renal Disease (MDRD) formula. The first group included patients with an eGFR below 70 mL/min/1.73 m^2^ (GFR-L group), while the second group included patients with an eGFR of 70 mL/min/1.73 m^2^ or above (GFR-H group). The eGFR threshold for subgroup differentiation arose from receiver operating characteristic (ROC) analyses, pinpointing the eGFR values that best-distinguished groups regarding FFR and non-hyperemic coronary assessment discrepancies. The distribution of patients in individual study groups and the number of analyzed vessels are presented in Fig. 1.

The decision to supplement the coronary angiography with a physiological assessment and the assessment method selection was conducted at the discretion of the operating clinician. This study used two systems for the physiological evaluation of coronary circulation: Abbott’s and Philips pressure wires. Intracoronary boluses of adenosine most often achieve hyperemia at a dose ranging from 100 µg to 400 µg. The non-hyperemic evaluations were conducted either via instantaneous wave-free ratio (iFR) or resting full-cycle ratio (RFR). Subsequent results were combined and reported as non-hyperemic assessments, irrespective of the exact method. A fractional flow reserve (FFR) value ≤0.80 and an iFR/RFR value ≤0.89 indicated ischemia.

The demographic attributes of the study cohorts, past medical history, concurrent conditions, assessed vessel locations, and physiological assessment outcomes were evaluated. Additionally, the compatibility of results using different assessment methods was analyzed. The study also explored factors affecting discrepancies in physiological evaluations across the two groups.

Ethical approval for this retrospective study was obtained from the ethics board of Jagiellonian University Medical College (Approval No: 1072.6120.257.2022, dated 16th Nov 2022). Therefore, patients did not sign a consent form to participate in the registry; they only consented to treatment in our department.

### Statistical Analysis

Categorical variables are depicted as numbers and percentages. Continuous variables are provided as the mean, standard deviation (SD), or median with the first and third quartiles (Q1–Q3). Differences between groups were compared using Student’s *t*-test for normally distributed continuous variables, while the Wilcoxon test was employed for non-normally distributed continuous variables. Pearson’s chi-squared test evaluated categorical variables. Univariable and multivariable logistic regression analyses were presented to identify predictors of discordant physiological assessment results of coronary circulation (specifically, FFR positive and iFR/RFR negative and vice versa). The multiple regression model included factors identified by the stepwise regression model with a *p*-value threshold (0.25 to enter, 0.1 to leave). Univariate analyses for factors included in various models were presented. ROC curves were constructed to discern the optimal eGFR cut-off values for predicting discrepancies between FFR and iFR/RFR results. The correlation between FFR and iFR/RFR across the two groups was assessed using Pearson’s correlation coefficients—a two-sided *p*-value less than 0.05 denoted statistical significance. JMP®, Version 17.1.0 (JMP Statistical Discovery, Cary, NC, USA) was used for all statistical analyses.

## 3. Results

Data on 279 patients were analyzed. Of these, 118 patients (42.3%) were diagnosed with an eGFR <70 mL/min/1.73 m^2^ and are categorized in the GFR-L group. Conversely, 161 patients (57.7%) had an eGFR of ≥70 mL/min/1.73 m^2^ and were subsequently referred to as the GFR-H group.

Demographic variations were evident between the groups. The GFR-H group predominantly consisted of younger males with a marginally reduced body mass index (BMI) compared to the GFR-L group. Medical history analysis highlighted a greater prevalence of diabetes mellitus and atrial fibrillation within the GFR-L group, while the GFR-H group had a significantly higher proportion of smokers (Table 1).

**Table 1.** **Baseline clinical characteristics of the study population**.

	Group		*p*-value
	GFR-L	GFR-H	
	118 (42.3%)	161 (57.7%)	
Age, years, median (Q1–Q3)	71 (64.75–79)	66 (59–72)	<0.0001
Gender, female, n (%)	38 (32.2)	32 (19.9)	0.0190
Height, cm, mean (SD)	169.2 (7.7)	171.9 (7.7)	0.0075
Weight, kg, median (Q1–Q3)	84.5 (74.0–95.0)	83.0 (71.0–94.0)	0.6094
BMI, kg/m^2^, median (Q1–Q3)	29.2 (25.6–32.7)	28.1 (24.5–30.8)	0.0412
Diabetes mellitus, n (%)	58 (49.2)	54 (33.5)	0.0086
Arterial hypertension, n (%)	108 (91.5)	134 (83.8)	0.0563
Atrial fibrillation, n (%)	37 (31.4)	18 (11.3)	<0.0001
Previous MI, n (%)	56 (47.5)	78 (48.5)	0.8702
Previous PCI, n (%)	57 (48.3)	84 (52.2)	0.5231
Previous CABG, n (%)	7 (6.0)	7 (4.4)	0.5382
PAD, n (%)	17 (14.5)	25 (15.5)	0.8186
Current smoker, n (%)	50 (42.4)	94 (58.4)	0.0082
COPD, n (%)	8 (6.8)	10 (6.2)	0.8340
Previous stroke/TIA, n (%)	15 (12.8)	12 (7.5)	0.1357
Dyslipidemia, n (%)	92 (78.0)	126 (78.3)	0.9531
LVEF, %, median (Q1–Q3)	52.5 (35–60)	52 (41–60)	0.3287
Radial access, n (%)	96 (81.4)	135 (83.9)	0.5854

Abbreviations: BMI, body mass index; CABG, coronary artery bypass grafting; COPD, chronic obstructive pulmonary disease; (e)GFR, (estimated) glomerular filtration rate; LVEF, left ventricle ejection fraction; MI, myocardial infarct; PAD, peripheral arterial disease; PCI, percutaneous coronary intervention; TIA, transient ischemic attack; L, indicates the group with an eGFR <70 mL/min/1.73 m^2^; H, indicates the group with an eGFR ≥70 mL/min/1.73 m^2^.

In the GFR-L group, 175 coronary vessels were examined (42%), while 242 vessels (58%) were reviewed in the GFR-H group. The left anterior descending branch of the left coronary artery (LAD) was the assessed vessel in approximately 60% of procedures, the frequency of which was very similar in both study groups.

The GFR-H group exhibited a notably lower FFR value for all evaluated vessels, though this did not equate to a disparity in the frequency of positive FFR results. Focusing solely on LAD assessments, both FFR metrics (continuous and binary) were comparable between the groups. In contrast, for non-LAD vessels, the GFR-H group revealed substantially reduced FFR values, with more vessels displaying significant constriction.

We do not observe significant differences between the study groups when analyzing the results of the non-hyperemic assessment—both for all the tested vessels and for the LAD and locations outside the LAD separately.

However, we observed that, in contrast to FFR, non-hyperemic methods confirmed the hemodynamic significance of stenoses in the GFR-L group slightly more often, whereas we observed an inverse relationship in the GFR-H group; here, hemodynamic significance was confirmed less often when using the non-hyperemic methods. Detailed data are presented in Table 2.

**Table 2.** **The results of vessel assessment in the study groups (per vessel)**.

		Group		*p*-value
		GFR-L	GFR-H	
		175 (42.0%)	242 (58.0%)	
Vessel assessed				
	LAD	106 (60.6%)	143 (59.1%)	0.7610
	non-LAD	69 (39.4%)	99 (40.9%)	
All vessels				
	FFR ≤0.80, n (%)	77 (44.0%)	123 (50.8%)	0.1685
	FFR, median (Q1–Q3)	0.83 (0.76–0.89)	0.80 (0.75–0.87)	0.0202
	iFR/RFR ≤0.89, n (%)	90 (51.4%)	104 (43.0%)	0.0876
	iFR/RFR, median (Q1–Q3)	0.89 (0.84–0.94)	0.90 (0.86–0.94)	0.2186
LAD				
	FFR ≤0.80, n (%)	62 (58.5%)	87 (60.8%)	0.7086
	FFR, median (Q1–Q3)	0.79 (0.75–0.85)	0.78 (0.73–0.84)	0.1609
	iFR/RFR ≤0.89, n (%)	66 (62.3%)	79 (55.2%)	0.2668
	iFR/RFR, median (Q1–Q3)	0.88 (0.84–0.92)	0.89 (0.84–0.93)	0.3300
Non-LAD				
	FFR ≤0.80, n (%)	15 (21.7%)	36 (36.4%)	0.0425
	FFR, median (Q1–Q3)	0.89 (0.82–0.94)	0.83 (0.78–0.90)	0.0058
	iFR/RFR ≤0.89, n (%)	24 (34.8%)	25 (25.3%)	0.1812
	iFR/RFR, median (Q1–Q3)	0.94 (0.86–0.98)	0.94 (0.89–0.97)	0.9125

Abbreviations: FFR, fractional flow reserve; (e)GFR, (estimated) glomerular filtration rate; iFR, instantaneous wave-free ratio; LAD, left anterior descending artery; RFR, resting full-cycle ratio; L, indicates the group with an eGFR <70 mL/min/1.73 m^2^; H, indicates the group with an eGFR ≥70 mL/min/1.73 m^2^.

**Fig. 2.**
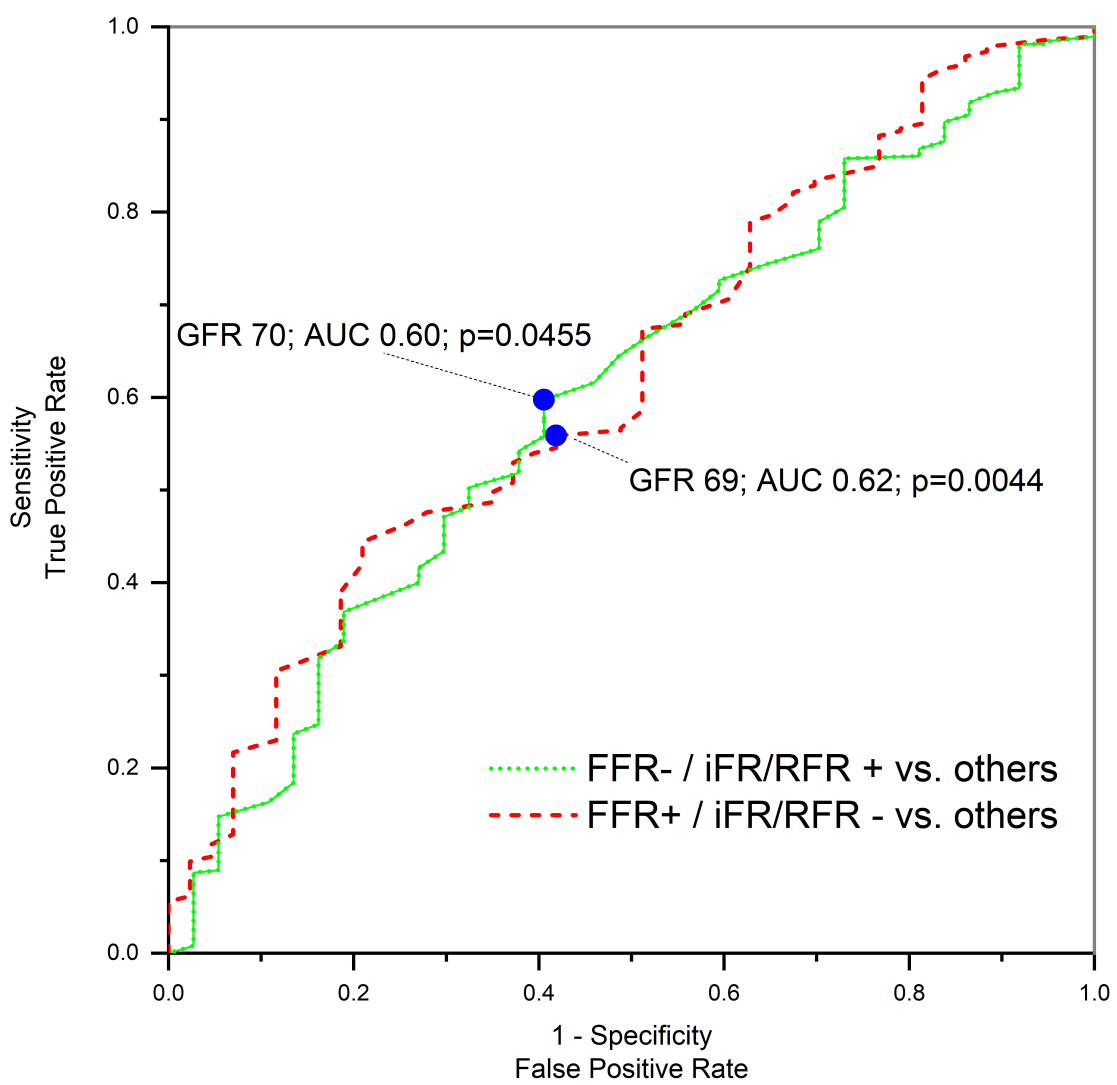
**ROC analysis of FFR | iFR/RFR discrepancies**. FFR, fractional flow reserve; GFR, glomerular filtration rate; iFR, instantaneous wave-free ratio; RFR, resting full-cycle ratio; AUC, area under the curve; ROC, receiver operating characteristic; +, positive assessment result; -, negative assessment result.

A closer examination of the discrepancies between FFR and non-hyperemic evaluations, contingent on the GFR value, facilitated the differentiation of patients into the two study cohorts. High eGFR patients commonly exhibited an FFR+ | iFR/RFR- mismatch, which declined with decreasing eGFR. Conversely, an FFR- | iFR/RFR+ discordance was observed more frequently. The odds ratio for eGFR (per 1 mL/min/1.73 m^2^) in determining FFR- | iFR/RFR+ was 0.98 (95% CI: 0.97–0.99), *p* = 0.0455 and for FFR+ | iFR/RFR- was 1.02 (95% CI: 1.01–1.03); *p* = 0.0044. Corresponding ROC analyses are illustrated in Fig. 2.

Further scrutiny into the coherence between hyperemic and non-hyperemic methods indicated a consistent discrepancy rate across both groups, independent of lesion location (LAD *vs*. non-LAD). However, segmenting the mismatches into FFR+ | iFR/RFR- and FFR- | iFR/RFR+ showcased a higher prevalence of negative FFR results accompanied by positive non-hyperemic results in the GFR-L group. Conversely, the GFR-H group more frequently exhibited positive FFR with negative non-hyperemic outcomes. These patterns persisted irrespective of lesion location, attaining statistical significance for all evaluated vessels and those beyond the LAD domain (Table 3).

**Table 3.** **The concordance of vessel assessment in the study groups (per vessel)**.

		Group		*p*-value
		GFR-L	GFR-H	
		175 (42.0%)	242 (58.0%)	
Concordance: general				
	Concordant	144 (82.3%)	193 (79.8%)	0.5167
	Discordant	31 (17.7%)	49 (20.3%)	
	FFR- | iFR/RFR-	76 (43.4%)	104 (43.0%)	0.0056
	FFR- | iFR/RFR+	22 (12.6%)	15 (6.2%)	
	FFR+ | iFR/RFR-	9 (5.1%)	34 (14.1%)	
	FFR+ | iFR/RFR+	68 (38.9%)	89 (36.8%)	
Concordance: LAD				
	Concordant	86 (81.1%)	113 (79.0%)	0.6810
	Discordant	20 (18.9%)	30 (21.0%)	
	FFR- | iFR/RFR-	32 (30.2%)	45 (31.5%)	0.4085
	FFR- | iFR/RFR+	12 (11.3%)	11 (7.7%)	
	FFR+ | iFR/RFR-	8 (7.6%)	19 (13.3%)	
	FFR+ | iFR/RFR+	54 (50.9%)	68 (47.6%)	
Concordance: non-LAD				
	Concordant	58 (84.1%)	80 (80.8%)	0.5884
	Discordant	11 (15.9%)	19 (19.2%)	
	FFR- | iFR/RFR-	44 (63.8%)	59 (59.6%)	0.0037
	FFR- | iFR/RFR+	10 (14.5%)	4 (4.0%)	
	FFR+ | iFR/RFR-	1 (1.5%)	15 (15.2%)	
	FFR+ | iFR/RFR+	14 (20.3%)	21 (21.2%)	

Abbreviations: FFR, fractional flow reserve; (e)GFR, (estimated) glomerular filtration rate; iFR, instantaneous wave-free ratio; LAD, left anterior descending artery; RFR, resting full-cycle ratio; +, positive assessment result; -, negative assessment result; L, indicates the group with an eGFR <70 mL/min/1.73 m^2^; H, indicates the group with an eGFR ≥70 mL/min/1.73 m^2^.

In evaluating clinical factors that might predispose to variations between FFR and non-hyperemic evaluations, we first considered any discrepancy. For the GFR-L group, COPD emerged as a crucial factor, escalating the risk of discrepancy four-fold. This association retained its significance in multivariate analysis. Within the GFR-H group, older age and arterial hypertension reduced the risk of discrepancy, with hypertension remaining significant even after multivariate adjustments.

Narrowing the focus to cases where FFR was positive but non-hyperemic results were negative—a pattern more prevalent in the GFR-H group—both arterial hypertension and insulin-treated diabetes markedly reduced the discrepancy risk for this cohort. The significance of insulin therapy persisted in the multivariate assessment. Conversely, for the GFR-L group, no such predisposing factors were identified for this particular discrepancy.

When examining instances of negative FFR paired with positive non-hyperemic results—a trend more evident in the GFR-L group—two key risk-enhancing factors surfaced for this cohort: insulin-treated diabetes (elevating risk over 11 times) and COPD (enhancing risk five-fold). Multivariate adjustments underline the importance of insulin therapy, amplifying the risk of discrepancy almost 13 times. For the GFR-H group, post-PCI status emerged as a discrepancy-risk booster, amplifying it fourfold. This association was also noted following multivariate analysis (Table 4).

**Table 4.** **Univariate and multivariate analysis of FFR | iFR/RFR discordance factors**.

		Group		Group	
		GFR-L	*p*-value	GFR-H	*p*-value
		OR (95% CI)		OR (95% CI)	
Univariate analysis: Predictors of FFR+ | iFR/RFR-					
	Hypertension (yes *vs*. no)	0.68 (0.08–5.86)	0.7255	0.36 (0.15–0.87)	0.0233
	DM treatment (insulin *vs*. others)	0.30 (0.03–2.69)	0.2819	0.20 (0.04–0.94)	0.0423
Multivariate analysis: Predictors of FFR+ | iFR/RFR-					
	Hypertension (yes *vs*. no)	-	-	0.48 (0.10–2.26)	0.3541
	DM treatment (insulin *vs*. others)	-	-	0.20 (0.04–0.94)	0.0429
Univariate analysis: Predictors of FFR- | iFR/RFR +					
	DM treatment (insulin *vs*. others)	11.56 (1.32–100.95)	0.0269	2.30 (0.48–11.00)	0.2973
	post PCI (yes *vs*. no)	0.82 (0.34–2.02)	0.6694	4.04 (1.11–14.68)	0.0343
	COPD (yes *vs*. no)	4.67 (1.40–15.57)	0.0120	2.03 (0.42–9.78)	0.3781
Multivariate analysis: Predictors of FFR- | iFR/RFR +					
	DM treatment (insulin *vs*. others)	12.96 (1.44–116.82)	0.0224	-	-
	post PCI (yes *vs*. no)	-	-	3.93 (1.08–14.34)	0.0385
	COPD (yes *vs*. no)	2.96 (0.23–37.44)	0.4014	1.71 (0.35–8.44)	0.5093
Univariate analysis: Predictors of overall FFR - iFR/RFR discordance					
	Age (per year)	1.01 (0.98–1.05)	0.4994	0.96 (0.93–0.99)	0.0414
	Hypertension (yes *vs*. no)	1.32 (0.28–6.21)	0.7268	0.38 (0.17–0.84)	0.0164
	COPD (yes *vs*. no)	4.05 (1.29–12.68)	0.0163	0.77 (0.21–2.79)	0.6950
Multivariate analysis: Predictors of overall FFR - iFR/RFR discordance					
	Age (per year)	1.01 (0.98–1.06)	0.4562	0.97 (0.94–1.01)	0.0954
	Hypertension (yes *vs*. no)	1.06 (0.22–5.10)	0.9420	0.43 (0.19–0.96)	0.0409
	COPD (yes *vs*. no)	4.11 (1.30–13.04)	0.0163	0.77 (0.21–2.86)	0.6998

Abbreviations: COPD, chronic obstructive pulmonary disease; DM, diabetes mellitus; FFR, fractional flow reserve; (e)GFR, (estimated) glomerular filtration rate; iFR, instantaneous wave-free ratio; OR, odds ratio; PCI, percutaneous coronary intervention; RFR, resting full-cycle ratio; CI, confidence interval; +, positive assessment result; -, negative assessment result; L, indicates the group with an eGFR <70 mL/min/1.73 m^2^; H, indicates the group with an eGFR ≥70 mL/min/1.73 m^2^.

**Fig. 3.**
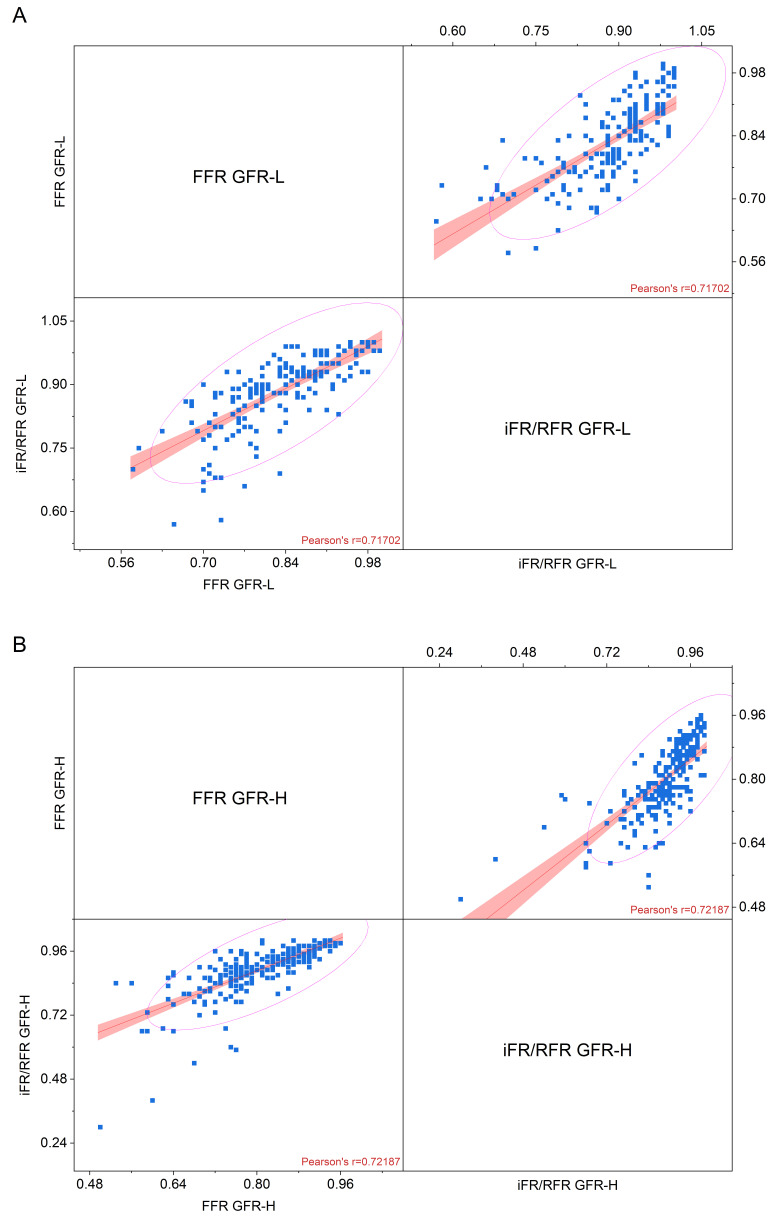
**Correlation between FFR and iFR/RFR in (A) GFR-L group and (B) GFR-H group of patients (linear fit, confidence bar, and confidence ellipse for prediction)**. FFR, fractional flow reserve; (e)GFR, (estimated) glomerular filtration rate; iFR, instantaneous wave-free ratio; RFR, resting full-cycle ratio; L, indicates the group with an eGFR <70 mL/min/1.73 m^2^; H, indicates the group with an eGFR ≥70 mL/min/1.73 m^2^.

Comparative analyses between hyperemic and non-hyperemic evaluation results in both cohorts yielded similar correlation magnitudes. A marginally superior Pearson correlation coefficient was noted in the GFR-H group: r = 0.717 for the GFR-L cohort versus r = 0.722 for the GFR-H group. A graphic image of the correlation is presented in Fig. 3.

## 4. Discussion

Our study performed several key findings:

(1) LAD consistently emerges as the most examined vessel in both cohorts. Notably, a strong concordance is observed between hyperemic and non-hyperemic evaluations. Both assessments identify LAD abnormalities with a similar rate of positivity: around 60% in each group. However, for vessels other than LAD, significant stenosis was identified within the GFR-L group in only about one in every five evaluations, marking a frequency roughly half of that observed in the GFR-H group.

(2) Individuals presenting lower eGFR values and undergoing physiological assessments of borderline stenoses in coronary arteries frequently exhibit negative FFR results, particularly when compared to those with higher eGFR values. This difference was manifested as a markedly higher prevalence of FFR- | iFR/RFR+ discrepancies within this cohort.

(3) Conversely, patients with higher baseline eGFR often demonstrate a higher frequency of FFR+ | iFR/RFR- discrepancies than those with diminished eGFR. An eGFR threshold of 70 mL/min/1.73 m^2^ emerged as the optimal discriminator for patients concerning the observed physiological discrepancies in coronary stenosis assessments.

The frequency of discrepancies in the hyperemic and non-hyperemic assessment in the presented group of patients was slightly less than 20%, comparable to the frequency published in the available literature [10]. The result of the non-hyperemic assessment and the location of the assessed lesions were similar in both groups. The influence of kidney function on physiological assessments has been the subject of several published studies. In one study conducted in Japan, researchers observed an increasing non-compliance rate in assessing FFR compared to RFR in patients with progressively lower GFR. This percentage was over 40% in patients with GFR below 30 mL/min/1.73 m^2^, and the correlation between FFR and RFR was the weakest in this group. Significantly, the discrepancy in the group of patients with the lowest GFR values results from a negative FFR value with a positive RFR result, which accounts for 3/4 of all discrepancies in this group [11]—an observation consistent with the results of the presented study. In turn, the correlation between the hyperemic and non-hyperemic assessment results in both study groups was slightly better, especially concerning the group of patients with GFR-L. However, in our case, the cut-off point for GFR was higher than in the case of the cited work.

Several factors were identified as discordant predictors of negative FFR and positive non-hyperemic testing. Among these factors were CKD and diabetes, as well as severe aortic stenosis, heart failure, and anemia [12]. The presented analyses confirm this relationship, especially for CKD and diabetes. They also identify factors such as COPD and a history of PCI that may significantly influence the discussed discrepancy.

Several reports show that younger patients are a factor that increases the risk of FFR positive and negative RFR [13, 14]. In the analyzed group, patients without CKD were characterized by a significantly lower age compared to the GFR-L group, and it is among younger patients that FFR+ | iFR/RFR- discordance predominates, consistent with published data. Interestingly, the presence of hypertension reduces the risk of this discrepancy.

In addition to demographic factors and comorbidities, a factor that may influence the results of the physiological assessment of coronary circulation may be a slightly broader background of changes occurring in the epicardial arteries in people with CKD compared to people without kidney disease. Indeed, the concept of CKD–mineral and bone disorder (CKD–MBD) appeared in the early 2000s, emphasizing the key role of kidney disease and the accompanying abnormalities in calcium, phosphate, hormonal (PTH), vitamin (D), and bone metabolism on the appearance of calcifications in the vessels [15].

Patients with CKD may, of course, present typical risk factors for the development of CAD, and their coronary circulation may show typical atherosclerotic lesions accompanied by calcifications in the intima. At the same time, coexisting kidney disease significantly impacts the appearance of a slightly different pathology both in the coronary circulation and in other arteries, namely the development of calcifications located in the media of the arteries [16]. In addition to CKD, arterial media calcifications may be related to diabetes or the patient’s age [17]. Calcifications located in the media of epicardial arteries usually do not cause narrowing of the artery lumen but increase its stiffness, thereby reducing coronary blood flow [18]. Therefore, their coexistence with typical atherosclerotic lesions may result in a more frequent FFR-/iFR/RFR+ discrepancy in patients with CKD and a more frequent occurrence of negative FFR results, which may result directly from reduced coronary blood flow. Since the above discrepancy is particularly visible in the studied population with CKD, in whom we may suspect a greater impact of CKD on the development of changes in coronary arteries than other typical CAD risk factors, the hypothesis that the medial calcifications influence the hyperemic assessment result seems particularly justified.

Factors influencing the development of CKD–MBD and cardiovascular complications in CKD patients are not limited to factors promoting the development of medial calcifications. Currently, many different mechanisms have been identified regarding the negative impact CKD has on the circulatory system. One of them is the increased activity of fibroblast growth factor-23 (FGF-23), which may independently impact the development of cardiac remodeling [19]. Other mechanisms include, for example, the activity of proteolytic enzymes, such as metalloproteinases (MPs). Published works indicate the relationship between the increased activity of several MPs on the development of arterial damage, including the development of aortic aneurysms, and, simultaneously, on kidney damage in various mechanisms, leading to the development of CKD [20, 21]. This diversity of pathomechanisms leading to coronary circulation dysfunction specific to the CKD patient population causes CAD in this group to have a slightly different nature than CAD in patients without CKD. Perhaps the discrepancies in the results of currently known and used methods of physiological assessment of coronary circulation result from the fact that the outcome of a given assessment method is influenced by the mechanism through which the functioning of the coronary circulation is impaired. However, it is necessary to conduct dedicated research to answer these specific questions.

## 5. Conclusions

CKD influences the outcomes of physiological evaluations of coronary circulation. Patients with diminished eGFR tend to yield negative results during hyperemic evaluations, especially concerning vessels other than the LAD. At the same time, non-hyperemic methods identify the examined lesions as hemodynamically significant relatively often, causing the FFR-/iFR/RFR+ discrepancy to occur frequently in the group of patients with lower eGFR values.

## Availability of Data and Materials

The datasets used and analyzed during the current study are available from the corresponding author upon reasonable request.
